# Protective Effect of Hesperidin on the Oxidative Stress Induced by an Exhausting Exercise in Intensively Trained Rats

**DOI:** 10.3390/nu11040783

**Published:** 2019-04-04

**Authors:** Sheila Estruel-Amades, Malén Massot-Cladera, Pau Garcia-Cerdà, Francisco J. Pérez-Cano, Àngels Franch, Margarida Castell, Mariona Camps-Bossacoma

**Affiliations:** 1 Secció de Fisiologia, Departament de Bioquímica i Fisiologia, Facultat de Farmàcia i Ciències de l’Alimentació, Universitat de Barcelona, 08028 Barcelona, Spain; sheilaestruel@ub.edu (S.E.-A.); malen.massot@ub.edu (M.M.-C.); paugc92@gmail.com (P.G.-C.); franciscoperez@ub.edu (F.J.P.-C.); angelsfranch@ub.edu (À.F.); marionacamps@ub.edu (M.C.-B.); 2 Institut de Recerca en Nutrició i Seguretat Alimentària (INSA-UB), Universitat de Barcelona, 08921 Santa Coloma de Gramenet, Spain

**Keywords:** antioxidant, catalase, exercise, glutathione peroxidase, hesperidin, oxidative stress, ROS, superoxide dismutase, training

## Abstract

Intensive exercise can lead to oxidative stress, which can be particularly deleterious for lymphoid tissues. Hesperidin has demonstrated its antioxidant activity, but few studies focus on its influence on intensive training. The aim of this study was to assess the impact of hesperidin on the oxidant/antioxidant status of lymphoid tissues after an intensive training program. Wistar rats were trained for five weeks (five days per week), including two exhaustion tests plus three trainings per week. During this period, animals were orally administrated with 200 mg/kg of hesperidin or vehicle (three days per week). The oxidative status was determined before, immediately after and 24 h after an additional exhaustion test. The production of reactive oxygen species (ROS) by peritoneal macrophages, superoxide dismutase (SOD) and catalase activities in spleen, thymus and liver, and hepatic glutathione peroxidase activity (GPx) were assessed. Hesperidin prevented an increase in ROS production induced by the additional exhaustion test. Likewise, hesperidin avoided a decrease in SOD and catalase activities in the thymus and spleen that was found after the additional exhaustion test. The antioxidant effects of hesperidin were associated with a higher performance in the assessed training model. These results suggest that hesperidin, acting as an antioxidant, can prevent oxidative stress induced by exercise and improve exercise performance.

## 1. Introduction

Reactive oxygen species (ROS) include a number of reactive molecules and free radicals derived from molecular oxygen as a result of normal cellular metabolism [1]. It is well known that low to moderate levels of ROS are necessary for many physiological processes in an organism, including gene transcription, signaling transduction, redox signal pathways and immune response [2,3]. However, the overproduction of ROS induces adverse effects on lipids, proteins and DNA that eventually lead to cell death. Under physiological conditions, ROS production is counterbalanced by both endogenous or externally supplemented antioxidants [1,3]. The main endogenous antioxidant enzymes are superoxide dismutase (SOD), catalase and glutathione peroxidase (GPx) [4]. SOD converts superoxide radicals into H_2_O_2_, which is catalyzed to H_2_O through the action of catalase and/or GPx. The latter enzyme can also be the donor of electrons to reduce peroxidase. While SOD and GPx are ubiquitously expressed [5], catalase is highly present in liver, kidney and red blood cells [6]. The fact that the membranes of immune cells are made of a high percentage of polyunsaturated fatty acids makes them highly sensitive to oxidative stress [7]. For that reason, since membrane-related signaling and gene expression are critical to maintaining immune cell functionality, they present high concentrations of antioxidant enzymes.

Oxidative stress occurs as a consequence of an imbalance between the production of ROS and antioxidant defense mechanisms [4]. As a result of this imbalance, a wide range of physiological processes can be altered, including immune function [8]. It is widely accepted that exercise, at any intensity, results in ROS synthesis, since it occurs naturally as a condition of oxidative metabolic processes [9,10]. Moderate exercise has been reported to increase ROS levels along with the body’s antioxidant defenses, contributing to the maintenance of a healthy oxidant status [11] and activating the immune system [12]. Such beneficial effects of moderate exercise seem to be due to the body’s adaptation to oxidative stress, increasing the expression and activity of endogenous antioxidant enzymes [4]. It has been reported that moderate exercise in mice induces a higher expression of catalase and GPx in intestinal lymphocytes [13], and induces higher catalase, SOD and GPx activities in the hippocampus of swimmer rats [14]. However, intensive exercise induces greater ROS production, overtaking the antioxidant system’s capacity and leading to oxidative stress [15]. A lot of studies have shown excessive ROS production due to strenuous exercise training in athletes [2,16,17]. The higher ROS production in intensive exercise, among other effects, can compromise muscular force and result in muscle damage, thus decreasing muscle performance [2]. In addition, intensive exercise can enhance glucocorticoid production, which will cause immune suppression [18,19]. The current knowledge about the effect of intensive training on antioxidant enzyme activities in different compartments is quite controversial. Some authors have reported that exhausting exercise decreases thymic and splenic catalase and SOD concentrations [20], as well as lowering blood SOD activity [21]. However, other authors have associated exhaustive exercise with higher gene expression and activity of antioxidant enzymes [22,23,24,25]. In any case, an adequate redox balance is essential for the functionality of the immune system, even in conditions of intensive and exhausting exercise.

Polyphenols are products of plant secondary metabolism which are associated with antioxidant activities as well as other beneficial health effects [26,27,28]. In this sense, polyphenol supplements can improve antioxidant defenses and decrease ROS production. An in vitro study demonstrated that total polyphenolic fraction of olive oil increased glutathione levels in HeLa and HepG2 cell cultures [29]. Similarly, an extract of apple peel or cocoa polyphenols produced an increase of GPx, SOD and catalase activities in liver and thymus in rodents [30,31]. In addition, the flavanol catechin increases glutathione reductase (GR) activity in mice immediately after downhill running, a typical eccentric exercise [32].

Hesperidin and its aglycone form, hesperetin, are a type of polyphenols found mainly in citrus fruits [33]. Besides having an antioxidant ability [34], they also exert beneficial biological activities in obesity [35], cancer [36] and in systemic and intestinal immunity [37,38]. The antioxidant activity of hesperidin has been observed in a rat model of pleurisy by reducing liver ROS production and increasing both catalase and SOD liver activities [39]. Furthermore, in older rats, hesperetin has been demonstrated to improve antioxidant status, mainly by increasing both catalase and GR activities in the liver [40]. Hesperidin also protects against oxidative damage induced by exercise through a decrease in lipid peroxidation in swimmer rats [41]. However, although in previous studies hesperidin has shown its effects in the immune system [37,38], there are no studies focused on the oxidative status in lymphoid tissues under intensive exercise-induced oxidative stress. For this reason, the aim of the present study is to assess the impact of hesperidin supplementation on the oxidant and antioxidant status of lymphoid tissues in rats after an intensive training program and after an additional exhaustion test.

## 2. Materials and Methods

### 2.1. Animals

All animal procedures performed in this study were approved by the Ethical Committee for Animal Experimentation of the University of Barcelona and the Catalonia Government (CEEA/UB ref. 464/16 and DAAM 9257, respectively).

A total of 64 three-week-old female Wistar rats were purchased from Envigo (Huntingdon, United Kingdom) and maintained in the animal facilities of the Faculty of Biology from the University of Barcelona. Experiments began after a 7-day adaptation period. Animals were housed in polycarbonate cages (3 rats per cage) in a controlled environment of temperature and humidity, in a 12/12 h light/dark cycle. Water and food were provided ad libitum.

### 2.2. Training Program

**Figure 1:**
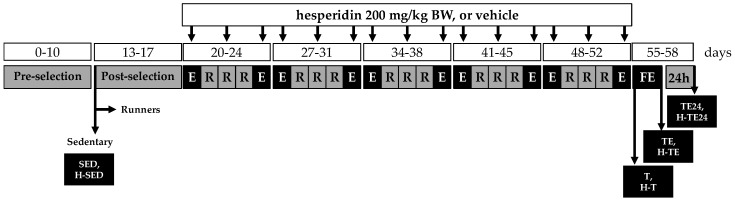
Experimental design. E = exhaustion test; FE = final exhaustion test; R = running training; T = vehicle-trained group; H-T = hesperidin-trained group; TE = T group with an additional exhaustion test; H-TE = H-T group with an additional exhaustion test; TE24 = TE group 24 h after the exhaustion test; H-TE24 = H-TE group 24 h after the exhaustion test.

Training was performed in two specialized treadmills for rodents: a LE8700 treadmill (Panlab, Harvard, USA) and an Exer3/6 treadmill (Columbus, OH, USA). Both devices allow the speed and exercise length to be controlled. The training program, based on that described by Batatinha et al. [42] and including 5 days per week of training, is summarized in Figure 1. The training program consisted of a preselection period (days 0–10), including 3 days of habituation on a turned-off treadmill, 2 days of resting (weekend) and 5 days in which the treadmill was turned on at a low but increasing speed (0–6 m/min) for an increasing length of time (15–20 min). At the end of this period, sedentary animals (two SED groups, *n* = 8 each one) were selected including those animals with low ability to run (4/16). Runner animals then began a 5-day period (post-selection period, days 13–17) in which animals ran once a day with increasing conditions of speed and duration (from 10 min/session at a speed of 5 m/min to 25 min/session at 25 m/min). In this period, 3/48 rats were excluded from the study because they were poor runners. Afterwards, the real training program began and lasted 5 weeks, from day 20 to 55 (Figure 1). In each of these 5 weeks, animals carried out an exhaustion test every Monday and Friday and ran for a limited and increasing time on Tuesday, Wednesday and Thursday. Each exhaustion test consisted of running 15 min at 60% of the previous Monday’s exhaustion test (the initial speed of the first Monday was 30 m/min), and the speed was progressively increased by 6 m/min every 2 min until exhaustion. The rats were considered exhausted when they could no longer maintain running. The maximum speed reached on Mondays was used as a reference for the following 3 days, running at 60% of such speed for 20, 25 and 30 min, on Tuesday, Wednesday and Thursday, respectively. After each run, the rats received a 50% solution of condensed milk (100 µL/100 g body weight (BW)) as a reward to positively reinforce the running. Sedentary animals also received this solution.

At day 55, once the 5-week training program was finished, runner animals were distributed into 3 groups, each one with a similar average in running ability (Figure 1). Two of them carried out an additional final exhaustion test. For this, animals ran for 15 min at 60% of the speed of the previous Monday’s exhaustion test, then the speed was increased 3 m/min every 2 min until the exhaustion of the animal.

Sedentary rats were exposed to the same conditions of maintaining, feeding and handling stress without training. BW and food intake were monitored throughout the study.

Oxygen consumption was recorded in representative animals using an OxyletPro system analyzer (Panlab, Harvard, MA, USA) integrated into the LE8700 treadmill. It was recorded in the pre-selection period (considered as basal values) and in every exhaustion test. Metabolism software (Panlab, Harvard, MA, USA) was used to calculate O_2_ at 20.8%, CO_2_ consumption (according to age, gender and weight of the animal) and the maximal respiratory exchange ratio (RER) (ratio between maximal CO_2_ consumption and maximal O_2_ consumption).

### 2.3. Hesperidin Supplement

From day 20 to 52 of the training program, a group of sedentary rats (H-SED group, *n* = 8) and a group of runner rats (*n* = 23, randomly selected at day 20 among runner rats) received a supplement of the flavanone hesperidin (5,7,3-trihydroxy-4-methoxyflavanone-7-rhamnoglucoside). The hesperidin was provided by Ferrer HealthTech (Murcia, Spain) and had a purity of 88.7% (92% S isomer) containing 5.1% isonaringin, 2.9% didymin, 0.2% hesperetin and 0.4% neohesperidin. The flavanone was administered by oral gavage three times per week at a dose of 200 mg/kg of rat BW. This dosage was chosen following a previous study demonstrating its beneficial effects on the immune system [37,38].

Animals that were not supplemented with hesperidin received the same volume of the vehicle (0.5% carboxymethylcellulose, 1 mL/100 g BW).

### 2.4. Animal Groups and Sample Collection

At day 56, in order to assess the oxidative stress at different time points, both hesperidin-supplemented (H-) and non-supplemented runner rats were distributed into three cohorts, each one with a similar average in the ability to run. The rats from the first cohort (T and H-T groups, *n* = 7–8 in each group) were euthanized 24 h after a regular training session (day 56). The animals from the second cohort (TE and H-TE groups, *n* = 7–8 in each group) were euthanized immediately after carrying out an additional final exhaustion test (day 57). The animals from the third cohort (TE24 and H-TE24 groups, *n* = 8 in each group) were euthanized 24 h after the additional exhaustion test to assess the long-term effects or the recovery (day 58). Likewise, hesperidin-supplemented and non-supplemented SED rats were euthanized randomly over the 3 days.

Animals were anaesthetized using ketamine (Merial Laboratories S.A., Barcelona, Spain)/xylazine (Bayer A.G., Leverkusen, Germany) and exsanguinated. Peritoneal macrophages were collected to assess ROS production. Spleen, thymus and gastrocnemius and soleus muscles were obtained and weighed. Afterwards, a part of the spleen, thymus and liver were frozen at ‒80 °C until processing to determine antioxidant activity.

### 2.5. Peritoneal Macrophage Isolation and ROS Production

Peritoneal macrophages were obtained, from five to six representative rats per group, as previously described [43]. Briefly, after the injection of 40 mL of sterile, cold phosphate-buffered saline (PBS) in the peritoneal cavity, a 2-min massage was performed and cells were collected. Once centrifuged (538× *g*, 10 min, 4 °C), cells were resuspended with cold Roswell Park Memorial Institute (RPMI) medium without phenol red, and supplemented with 1% heat-inactivated fetal bovine serum, 100 IU/mL streptomycin-penicillin, 2 mM L-glutamine and 0.05 mM 2-mercaptoethanol (Sigma-Aldrich, Madrid, Spain). Finally, macrophage counts were assessed by a Spincell hematology analyzer (MonLab Laboratories, Barcelona, Spain) properly calibrated for this type of cell.

ROS production was quantified as previously established [43]. In brief, macrophages were plated (10^5^ cells/well) and incubated overnight. Afterwards, the macrophages were washed using warm RMPI medium and incubated for 30 min with 20 µM of reduced 2′,7′-dichlorofluorescein diacetate probe (H_2_DCF-DA, Invitrogen, Paisley, UK). Macrophage-derived ROS oxidized H_2_DCF-DA to a fluorescent compound (2′,7′-dichlorofluorescein), which was quantified by the fluorimeter Modulus^®^ Microplate Multimode Reader (excitation 538 nm, emission 485 nm, Turner BioSystems, CA, USA). ROS data were expressed as the area under the curve (AUC) between 0 and 150 min.

### 2.6. Catalase Activity in Spleen, Thymus and Liver

Spleen, thymus and liver samples were homogenized in 50 mM of potassium phosphate buffer containing 1 mM of EDTA-Na_2_ (pH 7.0), using a Polytron^®^ PT 10–35 (Kinematica AG, Lucerne, Switzerland), then centrifuged (10,000× *g*, 10 min, 4 °C). The spleen, thymus and liver supernatants were mixed with an appropriate volume of 0.036% H_2_O_2_ solution, and immediately the absorbance was read at 240 nm (every 10 s for 4 min) using a spectrophotometer (UV-160A, Shimadzu Corporation).

Catalase activity was calculated based on the rate of decomposition of H_2_O_2_, which was proportional to the reduction in the absorbance. Catalase activity was expressed as units of catalase per g of tissue (U/g).

### 2.7. SOD Activity in Spleen, Thymus and Liver

SOD activity was measured in the same spleen, thymus and liver homogenates used for catalase determination via the xanthine–xanthine oxidase method. Homogenates were mixed with xanthine oxidase, xanthine and cytochrome C. The xanthine and xanthine oxidase system generated a superoxide, which reduced cytochrome C. The reduction of cytochrome C was monitored spectrophotometrically at 550 nm (every 1 min for 5 min). One unit of SOD is defined as the amount of enzyme needed to exhibit 50% dismutation of the superoxide radical. SOD activity was expressed as U/g of tissue.

### 2.8. Glutathione Peroxidase Activity in Liver

GPx activity was quantified by the Colorimetric Assay Kit (Biovision, Milpitas, CA, USA) following the manufacturer’s protocol. Briefly, liver samples were homogenized with the assay buffer, using the Polytron^®^ PT 10–35. After centrifugation (10,000× *g*, 15 min, 4 °C), supernatants were incubated with the reaction mix containing the NADPH, GR and reduced glutathione solutions for 15 min to deplete oxidized glutathione of the sample. Afterwards, cumene hydroperoxide was added to start the reaction, and the absorbance was read at 340 nm immediately and 5 min later. Results are expressed as AU/g of tissue.

### 2.9. Statistical Analysis

Analysis of the data was carried out using the IBM Social Sciences software program (SPSS, version 22.0, Chicago, IL, USA). After confirming the equality and normality of the data by Levene’s and Shapiro−Wilk’s tests, respectively, a two-way ANOVA test was applied. When significant differences were detected, Tukey’s post hoc test was performed. A Kruskal−Wallis test was used when results were neither equally nor normally distributed, followed by a Mann–Whitney U test in the case of significant differences among groups. To compare variables during the study (e.g., maximum distance in the exhaustion tests), a repeated-measures ANOVA was applied followed by Student’s *t*-test. Significant differences were considered when *p* ≤ 0.05. With regard to multiple comparisons (Student’s *t*-test), *p* value was corrected, dividing it by the number of applied tests (Bonferroni correction). 

## 3. Results

### 3.1. Training Program

**Figure 2:**
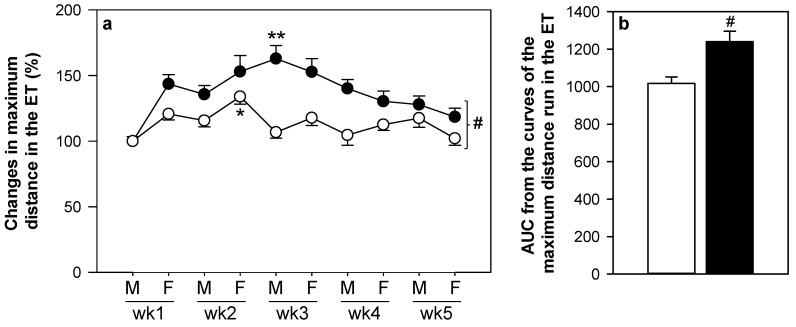
Changes in maximum distance run in the exhaustion tests throughout the study in percentage in comparison with the first day (**a**), and AUC from these curves showing changes in maximum distance run in the exhaustion tests (**b**). AUC = area under the curve, ET = exhaustion test, M = Monday, F = Friday, wk = week. The non-supplemented group is represented by white symbols and bars, and the hesperidin group by black symbols and bars. Data are expressed as mean ± SEM (*n* = 22–23). Statistical difference: * *p* < 0.001 in the non-supplemented group vs. the first and the last day (peak of performance); ** *p* < 0.001 in the hesperidin group vs. the first and the last day (peak of performance); # *p* < 0.005 between non-supplemented and the hesperidin-supplemented groups (Student’s *t*-test).

The training program lasted five weeks, in which animals ran an exhaustion test every Monday and Friday. The distance in the first exhaustion test was 898.17 ± 32.57 m (mean ± SEM) and 814.43 ± 34.20 for non-supplemented and hesperidin-supplemented animals, respectively. The distance run for each rat in the first exhaustion test was considered as 100% and, therefore, the time-course of the exercise performance in the five weeks of the training program was expressed accordingly (Figure 2a). The exercise performance of non-supplemented animals increased gradually up to the Friday of week two (*p* < 0.001), when rats ran about 134% in comparison with the first exhaustion test. Thereafter, performance decreased and ranged between 102% and 127% compared with the first exhaustion test (Monday, week one).

Hesperidin-supplemented animals showed a better performance than non-supplemented animals (*p* < 0.005). In fact, the highest performance of hesperidin-supplemented rats was achieved on the Monday of week three, when rats ran about 158% in comparison with the first exhaustion test (*p* < 0.001). Later, performance decreased and ranged between 121% and 152% compared to the first exhaustion test. The higher performance in the hesperidin-supplemented rats was also observed when considering the AUC throughout the study (Figure 2b). In addition, the absolute distance ran for hesperidin-supplemented and non-supplemented animals is summarized in Supplementary Figure S1, which also shows a higher performance in those hesperidin-supplemented animals in the middle of the study.

RER values throughout the exhaustion tests showed values around 1, meaning that the intensive training applied was mainly aerobic and neither exercise nor hesperidin-supplementation influenced O_2_ uptake.

In spite of these previous results, the final additional exhaustion test performed by the TE, H-TE, TE24 and H-TE24 groups showed no differences between interventions: non-supplemented animals ran a maximum distance of 1419.3 ± 100.0 m, while hesperidin-supplemented rats ran 1474.9 ± 138.7 m.

### 3.2. Body Weight, Food Intake and Organ Weight

No differences were observed in BW resulting from the hesperidin administration at the end of the intensive training program (day 52). However, both hesperidin- and non-supplemented runner animals showed higher BW compared with the corresponding SED group (Table 1). Regarding food intake, no differences were observed due to exercise training or hesperidin supplementation (Table 1).

**Figure 3:**
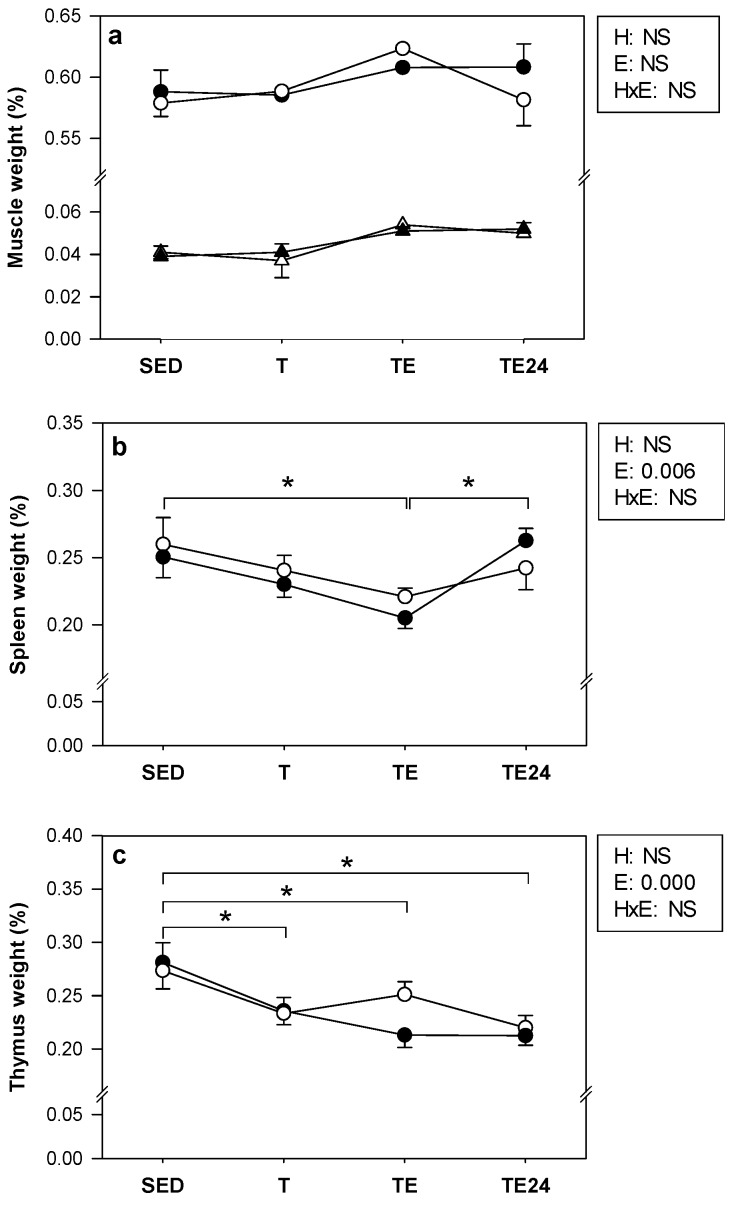
Relative weight of gastrocnemius muscle (**a**, circles), soleus muscle (**a**, triangles), spleen (**b**) and thymus (**c**) at the end of the study. The non-supplemented groups are represented by white symbols (⚪) and the hesperidin groups by black symbols (⚫). SED = sedentary groups, T = trained groups, TE = T groups with an additional exhaustion test, TE24 = TE groups 24 h after the exhaustion test, NS = no statistically significant differences detected. Data are expressed as mean ± SEM (*n* = 7–8). Statistical difference: the inset table shows two-way ANOVA results when applied (H, hesperidin; E, exercise, HxE, interaction between hesperidin and exercise). * *p* < 0.05 between exercise conditions for both the non-supplemented and the hesperidin-supplemented groups.

At the end of the study, the relative weight of the gastrocnemius and soleus muscles, spleen and thymus were considered (Figure 3). No changes were detected in either muscle’s relative weight from the exercise training or hesperidin administration. In the case of the spleen, hesperidin did not affect its weight. However, the TE condition caused a decrease in the spleen relative weight with respect to SED and TE24 animals. Regarding the weight of the thymus, T, TE and TE24 had a lower thymic weight compared to the SED animals, both in hesperidin- and non-supplemented animals, without any effect as a result of hesperidin administration being observed.

### 3.3. ROS Production by Peritoneal Macrophages

**Figure 4:**
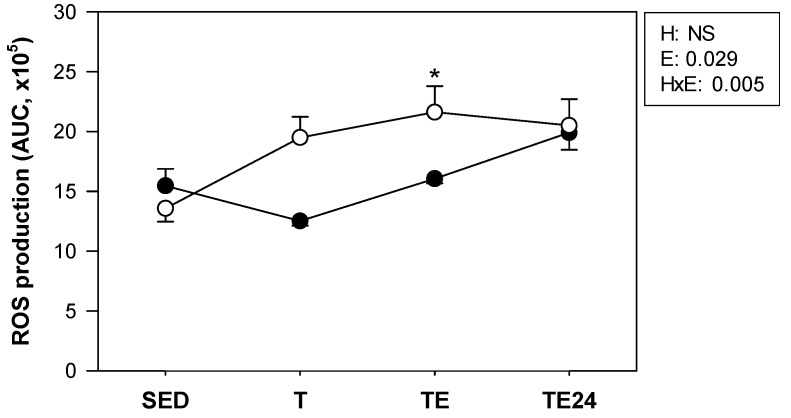
Production of ROS by peritoneal macrophages at the end of the study (AUC from 0 to 150 min). The non-supplemented groups are represented by white symbols (⚪) and the hesperidin groups by black symbols (⚫). SED = sedentary group, T = trained group, TE = T group with an additional exhaustion test, TE24 = TE group 24 h after the exhaustion test, NS = no statistically significant differences detected. Data are expressed as mean ± SEM (*n* = 5–6). Statistical difference: the inset table shows two-way ANOVA results when applied (H, hesperidin; E, exercise; HxE, interaction between hesperidin and exercise). * *p* < 0.05 vs. the SED group just in the non-supplemented animals.

ROS production tended to be higher in the non-supplemented trained animals (Figure 4), the significant difference being reached immediately after the additional final exhaustion test in the non-supplemented animals. This increase was prevented by the hesperidin administration.

### 3.4. SOD Activity

**Figure 5:**
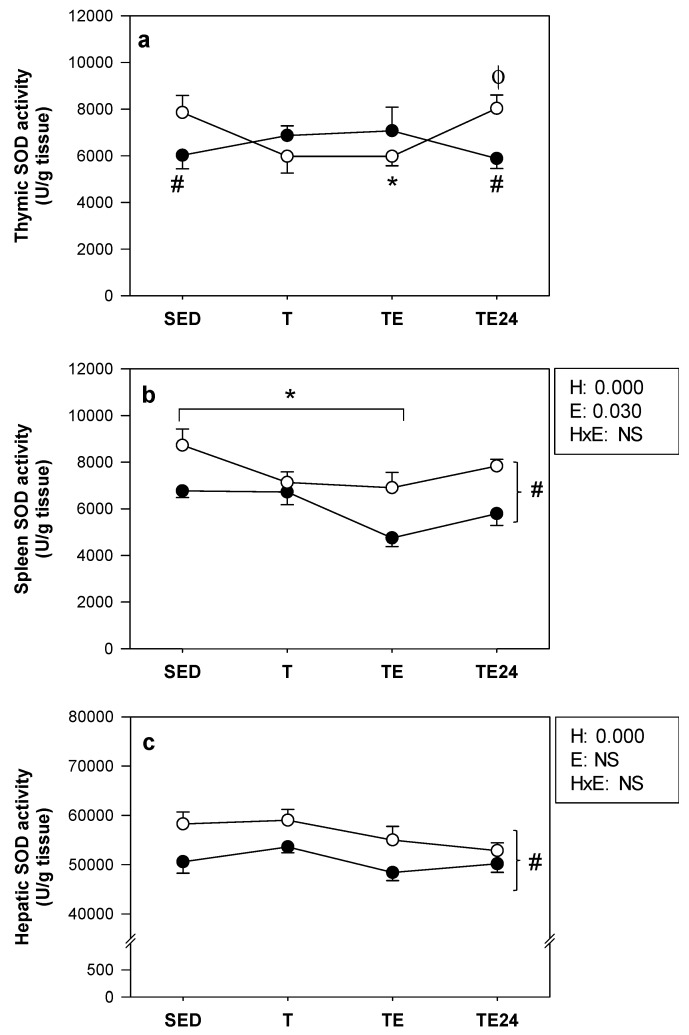
Superoxide dismutase (SOD) activity in the thymus (**a**), spleen (**b**) and liver (**c**) at the end of the study. The non-supplemented groups are represented by white symbols (⚪) and the hesperidin groups by black symbols (⚫). SED = sedentary group, T = trained group, TE = T group with an additional exhaustion test, TE24 = TE group 24 h after the exhaustion test, NS = no statistically significant differences detected. Data are expressed as mean ± SEM (*n* = 7–8). Statistical difference in (**a**) (Mann–Whitney U test): # *p* < 0.05 between non-supplemented and the hesperidin-supplemented groups; * *p* < 0.05 vs. SED group just in the non-supplemented animals; ϕ *p* < 0.05 vs. T group just in non-supplemented animals. Statistical difference in (**b**,**c**): the inset table shows two-way ANOVA results when applied (H, hesperidin; E, exercise; HxE, interaction between hesperidin and exercise); # *p* < 0.05 between non-supplemented and the hesperidin-supplemented groups; * *p* < 0.05 vs. exercise conditions.

SOD activity detected in the thymus, spleen and liver is summarized in Figure 5. In non-supplemented animals, a decrease in thymic SOD activity was detected in the TE group compared with the SED group, which returned to basal levels after 24 h. The hesperidin supplementation resulted in lower SOD activity in the H-SED and H-TE24 groups in comparison with their respective non-supplemented groups (Figure 5a).

Exercise training caused a reduction in spleen SOD activity just after the additional final exhaustion test in both hesperidin-supplemented and non-supplemented animals compared to the SED groups. Moreover, hesperidin administration decreased such activity with respect to the non-supplemented groups (Figure 5b).

Regarding the liver, exercise training did not modify SOD concentration. However, hesperidin administration resulted in lower SOD activity in comparison with the non-supplemented groups (Figure 5c).

### 3.5. Catalase Activity

**Figure 6:**
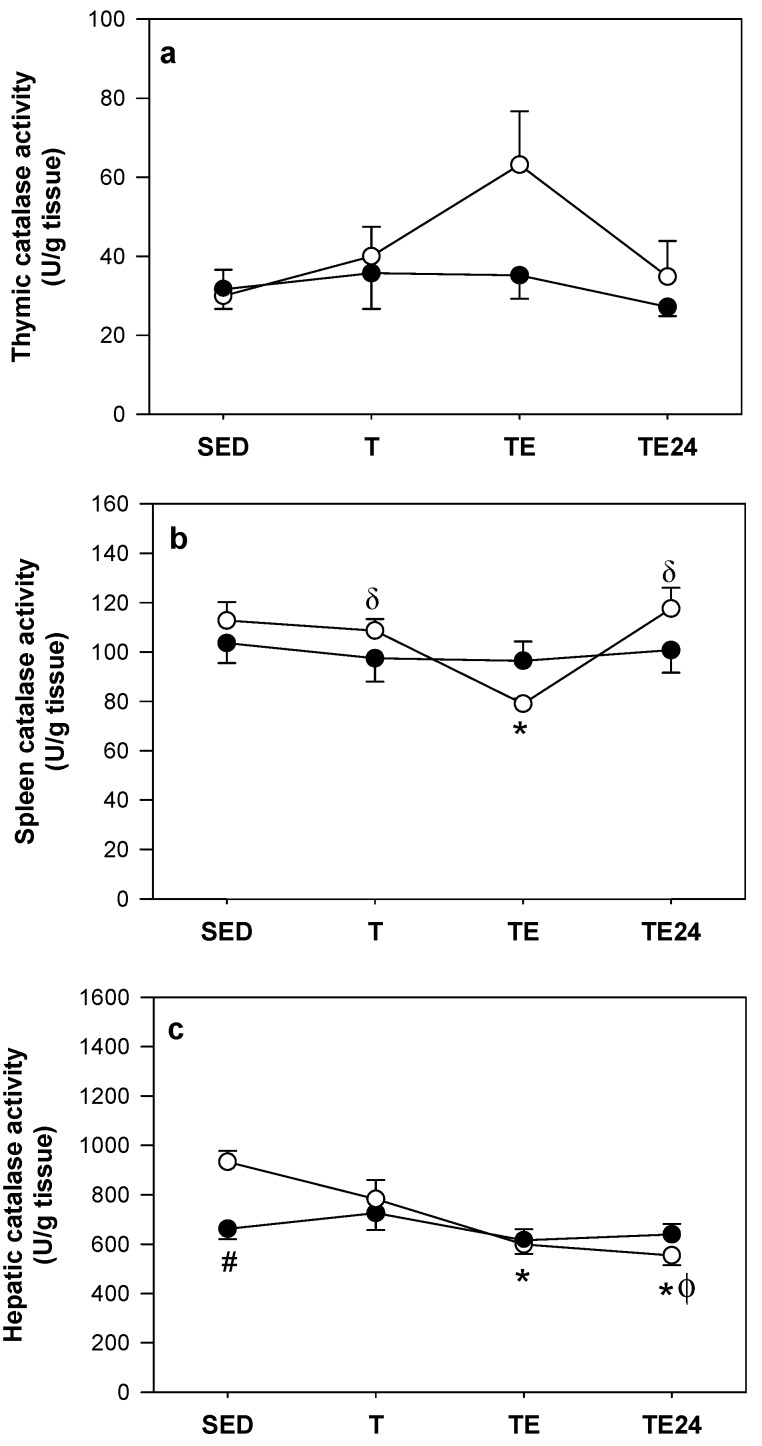
Catalase activity in the thymus (**a**), spleen (**b**) and liver (**c**) at the end of the study. The non-supplemented groups are represented by white symbols (⚪) and the hesperidin groups by black symbols (⚫). SED = sedentary group, T = trained group, TE = T group with an additional exhaustion test, TE24 = TE group 24 h after the exhaustion test. Data are expressed as mean ± SEM (*n* = 7–8). Statistical differences (Mann–Whitney U test): # *p* < 0.05 between non-supplemented and the hesperidin-supplemented groups; * *p* < 0.05 vs. SED group just in the non-supplemented animals; δ *p* < 0.05 vs. TE group just in the non-supplemented animals; φ *p* < 0.05 vs. T group just in the non-supplemented animals.

No significant changes were detected in thymic catalase activity due to either training, the final exhaustion test or hesperidin administration (Figure 6a).

In non-supplemented animals there was a marked drop in splenic catalase activity immediately after the additional final exhaustion test (Figure 6b). This activity was reestablished 24 h later. Hesperidin administration avoided these changes in splenic catalase activity.

The non-supplemented animals that followed the additional final exhaustion test (TE group) presented a decreased hepatic catalase activity with respect to the SED animals. This drop was maintained for at least 24 h (Figure 6c). Hesperidin administration prevented changes due to exercise, but the hepatic catalase activity in SED rats was lower than that in the non-supplemented SED group.

### 3.6. Hepatic Glutathione Peroxidase Activity

**Figure 7:**
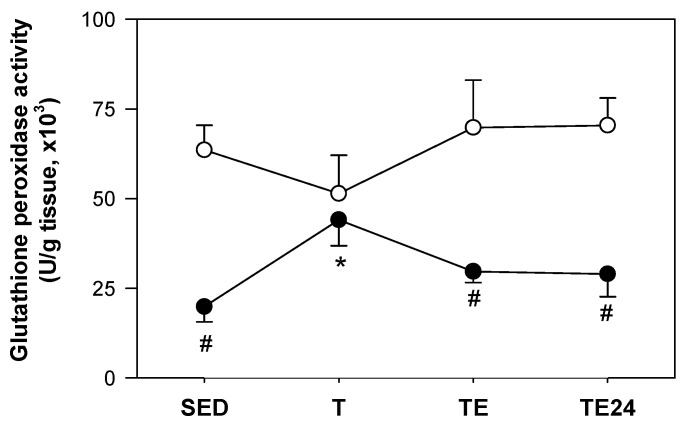
Hepatic glutathione peroxidase (GPx) activity at the end of the study. The non-supplemented groups are represented by white symbols (⚪) and the hesperidin groups by black symbols (⚫). SED = sedentary group, T = trained group, TE = T group with an additional exhaustion test, TE24 = TE group 24 h after the exhaustion test. Data are expressed as mean ± SEM (*n* = 6–8). Statistical difference (Mann–Whitney U test): # *p* < 0.05 between non-supplemented and the hesperidin-supplemented groups; * *p* < 0.05 vs. SED group just in the hesperidin animals.

There were no significant changes in hepatic GPx activity in non-supplemented animals resulting from training or the additional final exhaustion test. Nevertheless, exercise training in hesperidin-supplemented animals (H-T group) showed significantly higher GPx activity than the H-SED group (Figure 7). On the other hand, hesperidin administration significantly reduced hepatic GPx activity compared to its respective non-supplemented group in all the considered experimental conditions, with the exception of the T group (Figure 7). 

## 4. Discussion

The current study demonstrates the effects of hesperidin supplementation on exercise performance in intensively trained rats, the endogenous antioxidant defenses in their lymphoid tissues and liver and the oxidative stress induced by an additional exhaustion test.

In particular, the administration of 200 mg/kg of hesperidin three times per week for five weeks prompted a better performance in the exhaustion tests carried out during the study in Wistar rats. This effect was more evident in the first four weeks and even hesperidin-supplemented animals achieved the maximum performance one week later, which was higher than that in non-supplemented animals. The amount of hesperidin used was based on previous studies [37,38] evidencing its immunomodulatory effects. This amount in rats could be transformed to a human dosage by taking into account the body surface area [44]; accordingly, 200 mg/rat kg would be equal to 32.43 mg/human kg, and given that 1 L of orange juice provides about 600 mg hesperidin [45], the hesperidin amount per week would be supplied by a total of 13 L of orange juice per week. In this sense, we proposed here to use hesperidin supplements as has been previously described [46]. Previous studies have reported that polyphenols, particularly flavonoids such as quercetin, positively influence exercise performance [47,48,49,50]. Additionally, a recent study in humans has demonstrated that supplementation with a hesperetin extract (hesperidin aglycone) for four weeks in trained men improves exercise performance as assessed by a time-trial test on a cycle ergometer [51]. In spite of the better performance obtained, we did not find differences in the gastrocnemius or soleus muscle weight as a result of either the intensive training or the hesperidin supplementation.

Intensive training, together with an additional exhaustion test, increased ROS production of peritoneal macrophages, which may saturate an organism’s antioxidant mechanisms leading to oxidative stress [1]. This increase was prevented when rats were supplemented with hesperidin during the training program, a fact that could be due to the scavenging activity of polyphenols [52] that are able to neutralize ROS, such as superoxide anions, hydroxyl radicals, peroxynitrite and nitric oxide radicals [33,53,54]. It is well established that a decrease in exercise performance may be related to higher ROS production. In particular, higher ROS synthesis in muscles can produce fiber damage, which could contribute to muscle fatigue [55]. In this sense, we can suggest that hesperidin, like other flavonoids [56], can improve exercise performance by preventing muscle ROS accumulation, as we observed in peritoneal macrophages. Further studies are needed to check muscle ROS production in order to determine the influence of hesperidin in muscular tissue.

Under physiological conditions, ROS excess is counterbalanced by endogenous or externally supplemented antioxidants. Overproduction of ROS can produce oxidative stress and, consequently, immune functions—among other physiological processes—will be impaired. This study aimed to assess the influence of hesperidin supplementation in the endogenous antioxidant systems of two lymphoid tissues, the thymus (primary lymphoid tissue) and the spleen (secondary lymphoid tissue). Regarding the thymus, where T-lymphocyte maturation and selection occurs [57], a decrease in the thymic weight due to the intensive-training was observed. This result is in line with those reported in rats following a swimming training program for 21 days [58], and also the result in mice after a high-intensity swimming exercise for four weeks [59]. These results could be associated with thymocyte apoptosis due to the chronic stress induced by exercise [60], and would have consequences on thymic immune function. Although thymocyte apoptosis has been associated with oxidative stress [20], we found that lower thymic weight was also present after hesperidin administration. Moreover, we have detected that thymic SOD activity decreased immediately after the additional final exhaustion test, which agrees with previously reported data [20]. These results suggested that this antioxidant enzyme would be depleted when the additional exhaustion test was carried out, although it quickly recovered 24 h later. Interestingly, hesperidin administration prevented such reduction induced by the exhaustion test, although SOD activity was lower in sedentary animals receiving this flavonoid and remained constant whenever exercise was performed. This result could reflect the reduced need for this endogenous enzyme when an exogenous antioxidant is provided, even in conditions of oxidative stress, as in the intensive training and the additional final exhaustion test applied here. 

The spleen is a secondary lymphoid organ acting as a reservoir of lymphocytes, where the immune response to antigens initiated [61]. Spleen weight was reduced in trained rats just after the additional final exhaustion test, being recovered 24 h later. These results agree with those of Yuan et al. [59], who found that excessive exercise in mice caused spleen atrophy. Both results are likely related to the splenic vasoconstriction described when exercising [62], which increases muscle blood flow and is associated with splenic lymphocyte mobilization [63] due sympathetic nervous system activation [64]. Changes in splenic weight were also observed in hesperidin-supplemented rats, meaning that blood redistribution also occurred even with the antioxidant supplement. Apart from changes in splenic weight, the additional final exhaustion test decreased spleen SOD and catalase activities. These SOD results agree with those reported in rats after eight weeks of swimming (1 h/day, 5 days/week) [65] and both SOD and catalase activity decreases in the spleen after acute exercise in mice [20]. Taken all together, this would indicate the depletion of antioxidant defenses in the spleen after certain exercises, which could affect the immune function of this lymphoid tissue. Nevertheless, these results do not agree with those reported in the lymphocytes of active males performing high-intensity interval training who increased their SOD, catalase and GPx activities [66]. When supplemented with hesperidin, both sedentary and intensively trained animals showed, overall, lower levels of spleen SOD activity than were observed in non-supplemented animals, with no changes in catalase activity. As occurred in the thymus, this effect of hesperidin could reflect the lower necessity of SOD when an antioxidant is externally provided.

Antioxidant enzyme activities were also assessed in the liver, with higher SOD, catalase and GPx activities than the thymus or spleen. The training program and additional final exhaustion test applied did not significantly modify the liver SOD and GPx activities. However, the exhaustion test decreased catalase, which did not recover after 24 h. Similar results regarding liver SOD and/or GPx activities have been reported in both aged and young rats after eight weeks of swimming exercise [67], and in endurance-trained rats in a treadmill either for seven [68] or eight weeks [69]. These results could demonstrate the high antioxidant potential of the liver, which is not affected even in long exercise programs. Results concerning liver catalase activity are controversial. Although we found that the additional final exhaustion test decreased such enzymatic activity, other authors reported no changes in rats trained by swimming exercise for four weeks [70]. Regarding the influence of flavanone intake on liver antioxidant potential, hesperidin supplementation for five weeks decreased SOD and GPx activities in almost all groups, and lowered catalase activity just in sedentary rats. On the contrary, it has been reported that hesperidin was able to increase liver antioxidant activities in rats when administered at doses of 20–80 mg/kg BW for 10 days [71], as well as when it was administered to streptozotocin-induced diabetic rats at doses of 25–50 mg/kg BW for 35 days [72]. The discrepancies between our hesperidin results and those reported may derive from the flavanone dose applied, which was higher in our case and able to counteract oxygen radicals by itself, and not by enhancing endogenous antioxidant defenses. It is postulated that flavonoids, and in particular hesperidin and hesperetin, exert their antioxidant effect through two main mechanisms: by a direct radical scavenging of ROS accumulation, thus preventing oxidative damage, and by enhancing defense of endogenous antioxidants, for example, by increasing antioxidant enzymatic activities [34]. In our case, the protective effect of hesperidin at 200 mg/kg BW (three times per week for five weeks) against oxidative damage, induced by an intensive exercise, was more likely the result of its ability to scavenge ROS overproduction, rather than its influence on enzymatic antioxidant activities.

Overall, we have demonstrated the effect of hesperidin supplementation on intensively trained rats. Although the amount of hesperidin used here was supplied by extracts rather than natural orange juice, further studies must define the minimum dose and the posology able to induce antioxidant effects. Moreover, the current results give cause to consider further research focused on the effects of hesperidin on the immunosuppression derived from intensive exercise.

## 5. Conclusions

Our results indicate that hesperidin supplementation in intensively trained rats improves exercise performance, maintains endogenous antioxidant defenses in lymphoid organs and the liver and prevents the oxidative stress induced by an additional exhaustion test.

## Figures and Tables

**Table 1:** Body weight of the animals at the beginning of the study, and before and after the five-week training program and hesperidin supplementation (day 20 and 52, respectively), and food intake before and after the five-week training program and hesperidin supplementation.

Group	Body Weight (g)			Food Intake (g/day/rat)	
	Day 0	Day 20	Day 52	Day 20	Day 52
SED	67.67 ± 3.41	144.80 ± 3.62	206.57 ± 8.22	15.76 ± 0.29	15.68 ± 0.10
H-SED			205.00 ± 4.46		15.77 ± 0.19
Run	71.85 ± 1.91	154.33 ± 1.59	222.17 ± 3.09 *	15.27 ± 0.21	14.86 ± 0.49
H-Run			221.65 ± 3.61 *		15.59 ± 0.45

SED = non-supplemented sedentary group; H-SED = hesperidin sedentary group; Run = non-supplemented runner condition; H-Run = hesperidin runner condition. Data are expressed as mean ± SEM (*n* = 8–23). Statistical difference: * *p* < 0.05 vs. the corresponding SED group (Student’s *t*-test).

## Supplementary Materials

The following are available online at https://www.mdpi.com/2072-6643/11/4/783/s1, Figure S1: Maximum distance run in the exhaustion tests throughout the study.
