# Supplementary material for: Protective Effect of Hesperidin on the Oxidative Stress Induced by an Exhausting Exercise in Intensively Trained Rats
Source: Nutrients. 2019 Apr 4;11(4):783. doi: 10.3390/nu11040783 (PMC6520900; doi:10.3390/nu11040783)

Supplementary material

Figure S1. Maximum distance run in the exhaustion tests throughout the study. M=Monday, F=Friday, wk=week. The non-supplemented group is represented by white symbols, and the hesperidin group by black symbols. Data are expressed as mean ± SEM (n=22−23). Statistical difference: *$p < 0.05$ between non-supplemented and the hesperidin-supplemented groups (Student's t-test).

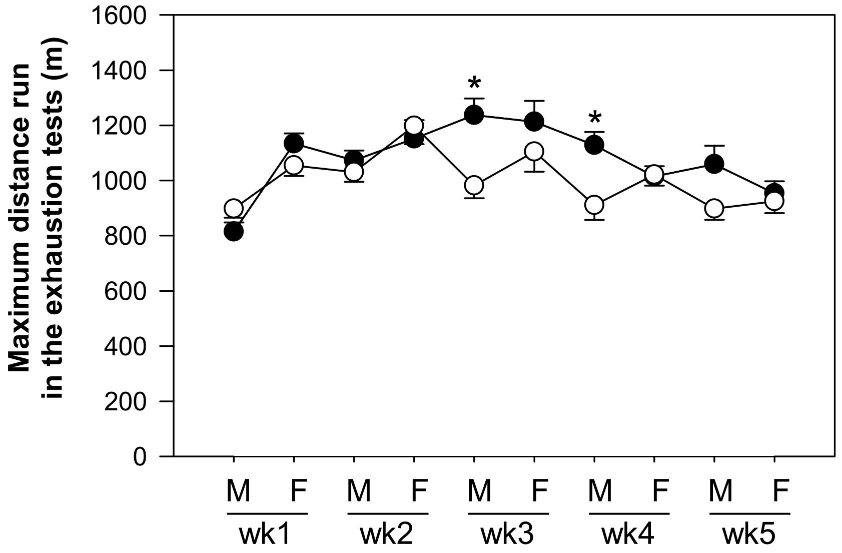

Supplement: Supplementary file 1 [file nutrients-11-00783-s001.pdf]
